# Machine Learning Reveals Distinct Phenotypic Virulence Clusters in *Candida* Isolates Recovered from the Nasopharynx of COVID-19 Convalescents

**DOI:** 10.3390/jof12100711

**Published:** 2026-09-22

**Authors:** Yekaterina Koloskova, Bakhyt Ramazanova, Kamilya Mustafina, Tolkyn Begadilova, Rustam Yussupov, Zamzagul Khandilla, Darya Bunyayeva, Azat Kalmyrzayev, Mohamed Ahmed Mabrouk, Akmaral Bissekenova

**Affiliations:** 1 Department of Microbiology, Virology, Asfendiyarov Kazakh National Medical University, 94 Tole bi Street, Almaty 050012, Kazakhstan; koloskova.e@kaznmu.kz (Y.K.); handilla.z@kaznmu.kz (Z.K.); darklight.90@mail.ru (A.K.);; 2 International Faculty of General Medicine, Asfendiyarov Kazakh National Medical University, 94 Tole bi Street, Almaty 050012, Kazakhstan; mohmabrouk28@gmail.com

**Keywords:** *Candida* spp., machine learning, virulence factors, phenotypic clustering, biofilm formation, antifungal susceptibility, Random Forest, SHAP, COVID-19 convalescent patients

## Abstract

**Background**: *Candida* spp. are opportunistic fungi whose pathogenic potential arises from interactions among multiple phenotypic traits that may not be captured by conventional single-factor analyses. This study aimed to identify multidimensional phenotypic virulence profiles among clinical *Candida* isolates using unsupervised machine learning. **Methods**: A total of 107 nasopharyngeal isolates recovered from COVID-19 convalescent patients in Almaty, Kazakhstan, during 2021–2022 were evaluated for biofilm formation, phospholipase, proteinase, hemolytic activities and susceptibility to fluconazole, voriconazole, itraconazole, and amphotericin B. Phenotypic data were analyzed using principal component analysis, Uniform Manifold Approximation and Projection, hierarchical clustering, permutational multivariate analysis of variance (PERMANOVA), Random Forest, and SHAP analysis. **Results**: *Candida albicans* predominated among the isolates (69.2%), and non-*albicans Candida* species accounted for 30.8%. Interspecies differences were observed in biofilm formation (*p* = 0.048) and itraconazole minimum inhibitory concentrations (*p* < 0.001). Three phenotypic clusters independent of species were identified (PERMANOVA: pseudo-F = 6.72, *p* = 0.0002). Across 20 repeated 5-fold cross-validation runs, the Random Forest model achieved an aggregated accuracy of 0.785, a mean balanced accuracy of 0.518 (95% CI: 0.461–0.577), and a macro-F1 score of 0.504 (95% CI: 0.444–0.559). SHAP identified itraconazole susceptibility, biofilm formation, and phospholipase activity as the strongest predictors. **Conclusions**: These findings demonstrate that integrating microbiological phenotyping with machine learning can reveal multidimensional virulence profiles beyond conventional species-based classification.

## 1. Introduction

*Candida* infections remain a major cause of fungal disease worldwide. Invasive candidiasis is associated with high morbidity and mortality among hospitalized patients and is caused by both *Candida albicans* and an expanding spectrum of non-*albicans Candida* (NAC) species exhibiting distinct virulence traits and antifungal susceptibility profiles [1]. Although most clinical *Candida* isolates initially exist as commensals colonizing mucosal surfaces, these colonizing strains are increasingly recognized as an important reservoir for subsequent invasive infection in immunocompromised patients [2,3]. This reservoir may be particularly relevant following viral respiratory infections when alterations in host immunity may facilitate opportunistic fungal colonization.

Current evidence indicates that the oral cavity and oropharynx constitute major reservoirs of microorganisms capable of colonizing the lower respiratory tract, thereby contributing to respiratory infections, particularly ventilator-associated pneumonia [4,5]. Furthermore, microbial biofilms rapidly develop on the surfaces of endotracheal tubes, providing an additional reservoir for opportunistic pathogens. Microorganisms detected in dental plaque and the oropharynx have frequently been shown to correspond to those recovered from endotracheal tubes and bronchoalveolar lavage fluid. Consequently, the nasopharynx should be regarded not only as a site of *Candida* spp. colonization but also as a potential reservoir of clinically relevant strains capable of contributing to infectious processes in the lower respiratory tract [6].

The virulence of *Candida* spp. is increasingly viewed as a multifactorial phenotype that emerges from dynamic interactions between the pathogen, the host, and the surrounding environment [7]. Contrary to the traditional view that virulence is determined primarily by species identity, accumulating evidence indicates considerable phenotypic heterogeneity even among clinical isolates of the same *Candida* species. Individual isolates may differ markedly in their capacities for adhesion, biofilm formation, hydrolytic enzyme production, and other virulence-associated traits, suggesting that pathogenic potential cannot be adequately inferred from species identification alone [8]. The expression of these virulence-associated traits is highly dynamic and is shaped by the anatomical niche of colonization, host immune status, and the clinical context of infection [9]. Furthermore, the *Candida* cell wall undergoes continuous remodeling in response to host-derived environmental stresses and antifungal pressure, directly orchestrating fungal adaptation, immune evasion, and the phenotypic expression of these virulence traits [10]. Ultimately, elevated expression of these factors contributes to the invasive potential of *Candida* strains and may facilitate the development of disseminated infection [11].

Because virulence is an emergent property, determining the pathogenicity of clinical *Candida* isolates requires characterizing their full phenotypic profile. For instance, key virulence-associated traits including adhesion, tissue invasion, persistence, and biofilm formation display considerable phenotypic heterogeneity even within a single species [12,13,14]. Even among isolates of the same species, substantial differences exist in their capacity to colonize both biotic and abiotic surfaces, including host tissues, indwelling medical devices, and hospital environmental surfaces [15]. Conventional analytical approaches evaluate these traits independently, limiting their ability to capture the complex interactions that collectively determine the pathogenic phenotype. Although biofilm formation, hydrolytic enzyme production, hemolytic activity, and antifungal susceptibility have been extensively investigated in *Candida* spp., these characteristics are typically examined as separate variables rather than integrated into a multidimensional phenotypic profile [16,17,18,19,20,21,22,23].

Machine learning provides a powerful framework for the integrative analysis of multidimensional phenotypic data by identifying complex nonlinear relationships without requiring predefined assumptions about their underlying structure. Specifically, advanced techniques such as Random Forests, Support Vector Machines (SVMs), and unsupervised clustering algorithms are gaining increasing prominence in modern healthcare. Within clinical microbiology, these approaches offer substantial advantages, ranging from uncovering hidden patterns in host–pathogen interactions to the accurate prediction of antifungal resistance and the phenotypic profiling of strains. Consequently, multiple virulence-associated traits can be integrated into a unified multidimensional virulence profile that more comprehensively characterizes the pathogenic potential of clinical *Candida* isolates [24,25,26,27,28,29].

Accordingly, the primary objective of this study was to identify and statistically validate latent multidimensional virulence clusters among nasopharyngeal *Candida* isolates recovered from COVID-19 convalescent patients using unsupervised machine learning.

## 2. Materials and Methods

### 2.1. Study Design

This cross-sectional laboratory-based microbiological study comprehensively characterized the phenotypic pathogenic potential of *Candida* isolates recovered from the nasopharynx of COVID-19 convalescent patients through the integrated analysis of virulence-associated traits, antifungal susceptibility profiles, and machine learning-based phenotypic clustering.

### 2.2. Ethical Approval

The study was approved by the Local Bioethics Committee of Asfendiyarov Kazakh National Medical University. Ethical approval for the collection of biological specimens from COVID-19 convalescent patients was granted under Protocol No. 13 (119) and dated 29 September 2021 (informed consent was obtained from all subjects involved in the study), and approval for the subsequent microbiological investigation was granted under Protocol No. 19 (155), dated 30 September 2024.

### 2.3. Study Population and Isolate Collection

*Candida* isolates were recovered from nasopharyngeal swabs collected from a consecutive, unselected cohort of COVID-19 convalescent patients between November 2021 and February 2022 as part of the “Health Assessment of COVID-19 Convalescent Patients” project. Swabs were routinely collected on the day of hospital discharge following a negative SARS-CoV-2 PCR test, irrespective of clinical signs of fungal infection. Because clinical examination for oral lesions was not included in the study protocol, these isolates reflect incidental nasopharyngeal colonization rather than verified oropharyngeal candidiasis. Additionally, because specimen acquisition was integrated into a standardized post-discharge screening protocol, granular patient-level clinical records were unavailable for individualized cohort stratification. Following primary isolation, pure cultures were deposited in the culture collection of the Department of Microbiology and Virology at Asfendiyarov Kazakh National Medical University and stored at −80 °C until further analysis.

### 2.4. Fungal Identification

Preliminary identification of yeast isolates was performed on CHROMagar™ Candida Plus medium (CHROMagar, Paris, France). Species-level identification was subsequently confirmed by matrix-assisted laser desorption/ionization time-of-flight mass spectrometry (MALDI-TOF MS) using a Microflex LT mass spectrometer (Bruker Daltonics, Bremen, Germany) at the Clinical Research Laboratory of Karaganda Medical University.

### 2.5. Antifungal Susceptibility Testing

Antifungal susceptibility testing of *Candida* isolates was performed using the broth microdilution method in 96-well microtiter plates in accordance with the CLSI M27 guidelines at the Clinical Investigated Laboratory of Karaganda Medical University. Twofold serial dilutions of fluconazole (Yuria-Pharm LLC, Kyiv, Ukraine), voriconazole (United Biotech (P) Ltd., Baghbania, India), itraconazole (Yuria-Pharm LLC, Kyiv, Ukraine), and amphotericin B (JSC Sintez, Kurgan, Russia) were prepared directly in the microtiter plates using a multichannel pipette.

RPMI 1640 medium (Elabscience, Houston, TX, USA) supplemented with L-glutamine, without sodium bicarbonate, and buffered to pH 7.0 with 0.165 M 3-(N-morpholino)propanesulfonic acid (MOPS) was used as the test medium. The inoculum was adjusted to a turbidity equivalent to a 0.5 McFarland standard (bioMérieux, Marcy-l’Étoile, France), and 100 μL of the standardized fungal suspension was added to each well. The plates were incubated at 35 °C for 48 h.

Minimum inhibitory concentrations (MICs) were determined visually against a black background. For the azole antifungal agents—fluconazole, voriconazole, and itraconazole—the MIC was defined as the lowest concentration resulting in at least a 50% reduction in visible growth relative to the drug-free growth control. For amphotericin B, the MIC was defined as the lowest concentration producing complete inhibition of visible growth.

Quality control was performed using the reference strain *Candida parapsilosis* ATCC 22019 (American Type Culture Collection, Manassas, VA, USA). The MIC values obtained for the quality-control strain were within the CLSI-recommended ranges, confirming the validity of the susceptibility testing procedure.

Antifungal susceptibility data were interpreted using a dual-standard framework in accordance with Clinical and Laboratory Standards Institute (CLSI) guidelines: Species-specific clinical breakpoints (CBPs) from CLSI supplement M60 were applied to fluconazole and voriconazole. For fluconazole, isolates of *C. albicans*, *C. tropicalis*, and *C. parapsilosis* were classified as susceptible (S) at MIC ≤ 2 µg/mL, susceptible dose-dependent (SDD) at 4 µg/mL, and resistant (R) at ≥8 µg/mL. *C. krusei* was designated as intrinsically resistant to fluconazole. For voriconazole, breakpoints for *C. albicans*, *C. tropicalis*, and *C. parapsilosis* were defined as S ≤ 0.125 µg/mL, SDD (0.25–0.5 µg/mL), and R ≥ 1 µg/mL, whereas criteria for *C. krusei* were S ≤ 0.5 µg/mL, SDD (1 µg/mL), and R (≥2 µg/mL).

Because formal clinical breakpoints are not established by CLSI for amphotericin B and itraconazole across *Candida* species, interpretations for these agents were guided by Epidemiological Cutoff Values (ECVs) from CLSI supplement M59 to distinguish wild-type (WT) from non-wild-type (NWT) phenotypes. For amphotericin B, an ECV of ≤2 µg/mL was applied across all evaluated species. For itraconazole, species-specific ECVs are defined as ≤0.125 µg/mL (*C. albicans*), ≤0.5 µg/mL (*C. parapsilosis* and *C. tropicalis*), and ≤1 µg/mL (*C. krusei*). Given the lack of consensus CBPs, raw continuous MIC values for itraconazole were directly utilized as numerical features for machine-learning modeling.

### 2.6. Virulence Factor Assessment

#### 2.6.1. Phospholipase Activity

Phospholipase activity was determined using the egg yolk agar plate method described by Price et al. (1982) [30]. To prepare a standardized inoculum, the isolates were subcultured on Sabouraud dextrose agar (HuanKai, Guangzhou, China) and incubated at 37 °C for 48–72 h. Yeast suspensions were then prepared from the resulting cultures and adjusted to a turbidity equivalent to a 2.0 McFarland standard.

The standardized suspensions were spot-inoculated onto the surface of Sabouraud dextrose agar supplemented with NaCl, CaCl_2_, and sterile egg yolk emulsion. The inoculated plates were incubated at 37 °C for 10 days.

After incubation, the colony diameter and the total diameter of the colony plus the surrounding precipitation zone were measured. Phospholipase activity was expressed as the Pz index, calculated using the following formula:

(1) $$Pz\;index=\frac{colony\;diameter}{total\;diameter\;of\;the\;colony+the\;precipitation\;zone}$$

Pz values < 1.0 were considered indicative of phospholipase production, with lower Pz values corresponding to higher enzymatic activity.

#### 2.6.2. Proteinase Activity

Proteinase activity was assessed using the bovine serum albumin (BSA) agar plate method described by Aoki et al. (1994) [31]. The assay medium consisted of Sabouraud dextrose agar supplemented with 1% BSA (Sigma-Aldrich, St. Louis, MO, USA) as the protein substrate. To prepare a standardized inoculum, the isolates were subcultured on Sabouraud dextrose agar and incubated at 37 °C for 48–72 h. Yeast suspensions were then prepared from the resulting cultures and adjusted to a turbidity equivalent to a 0.5 McFarland standard. Subsequently, 10 μL of the standardized inoculum was spot-inoculated onto the surface of the BSA agar. The inoculated plates were incubated at 37 °C for 7 days. Following incubation, the plates were treated with 20% trichloroacetic acid to terminate proteolytic activity and subsequently stained with 1.25% amido black. Proteinase activity was assessed by the presence of a clear zone of proteolysis surrounding the colony and was expressed as the proteinase index (Prz), calculated as the ratio of the colony diameter to the total diameter of the colony plus the surrounding proteolytic zone:

(2) $$Prz\;index=\frac{colony\;diameter}{total\;diameter\;of\;the\;colony+the\;precipitation\;zone}$$

Prz values < 1.0 were considered indicative of proteinase production. For descriptive purposes, activity levels were classified using the same criteria as those applied to phospholipase activity.

#### 2.6.3. Hemolytic Activity

Hemolytic activity was assessed using the modified method of Manns et al. (1994), as described by Luo et al. (2001) [32,33]. The assay was performed on Sabouraud dextrose agar supplemented with 7% sheep erythrocytes and 3% glucose. To prepare a standardized inoculum, the isolates were subcultured on Sabouraud dextrose agar and incubated at 37 °C for 18 h. Yeast suspensions were then prepared from the resulting cultures and adjusted to a final concentration of 1 × 10^8^ cells/mL.

The standardized suspensions were spot-inoculated onto the surface of the agar medium. The inoculated plates were incubated at 37 °C in an atmosphere containing 5% CO_2_ for 48 h.

Following incubation, hemolytic activity was assessed by the presence of a hemolytic zone surrounding the colony and was expressed as the hemolytic index (Hz), calculated as the ratio of the colony diameter to the total diameter of the colony plus the surrounding hemolytic zone:

(3) $$Hz\;index=\frac{colony\;diameter}{total\;diameter\;of\;the\;colony+the\;precipitation\;zone}$$

Hz values < 1.0 were considered indicative of hemolytic activity. For descriptive analysis, activity levels were classified according to the same criteria as those used for phospholipase activity.

#### 2.6.4. Biofilm Formation

Biofilm formation was assessed using a crystal violet microtiter plate assay adapted from Gulati et al. (2018) [34]. Briefly, *Candida* suspensions were inoculated into 96-well flat-bottom polystyrene microplates (Sarstedt, Inc., Newton, NC, USA) at a final concentration of 10^6^ cell/mL in Sabouraud broth, with a final volume of 100 µL per well. To ensure technical precision and account for well-to-well variability, all assays were performed in technical triplicates for each clinical isolate using a standardized initial inoculum. Uninoculated Sabouraud dextrose broth (HiMedia Laboratories, Mumbai, India) served as a negative control on each microtiter plate. Plates were incubated at 37 °C for 24 h.

Following incubation, planktonic cells were removed and the wells were washed 3 times with PBS (pH = 7.2). The remaining adherent biofilms were fixed by air-drying for 30 min at room temperature and subsequently stained with 150 µL of 0.1% aqueous crystal violet solution for 15 min. Excess stain was removed by washing the wells three times with sterile distilled water, followed by air-drying. The biofilm-bound dye was solubilized by adding 150 µL of 96% ethanol per well and incubating for 15 min under gentle agitation. Optical density was measured at 620 nm using a Multiskan GO microplate reader (Thermo Fisher Scientific, Waltham, MA, USA). The optical density cut-off value (OD_c_) was calculated as the mean optical density of the negative controls plus three standard deviations (OD_c_ = mean OD_negative_ + 3SD_negative_).

Biofilm-forming ability was classified according to the criteria proposed by Stepanović et al. (2007): non-biofilm producer (OD ≤ OD_c_), weak biofilm producer (OD_c_ < OD ≤ 2 × OD_c_), moderate biofilm producer (2 × OD_c_ < OD ≤ 4 × OD_c_), and strong biofilm producer (OD > 4 × OD_c_) [35]:

For each isolate, the normalized continuous biofilm index was calculated by dividing the mean optical density of the test strain (averaged across technical triplicates) by the optical density cut-off value:

(4) $$Biofilm\;index=\frac{{\bar{OD}}_{strain}\;}{ODc}$$

The resulting index was used as a continuous variable in multivariate statistical analyses and machine-learning models. For descriptive characterization of biofilm formation, biofilm index values were interpreted using the corresponding thresholds: ≤1, no biofilm formation; >1 to 2, weak biofilm formation; >2 to 4, moderate biofilm formation; and >4, strong biofilm formation.

### 2.7. Statistical Analysis

Statistical analyses and machine-learning modeling were performed using Python (version 3.10) in the Google Colaboratory environment (Google LLC, Mountain View, CA, USA) with the pandas 2.2.3, NumPy 2.1.3, SciPy 1.16.3, statsmodels 0.15.0, scikit-learn 1.6.1, SHAP 0.52.0, UMAP-learn 0.5.12, and Matplotlib 3.10.0 libraries [36].

Categorical variables were summarized as frequencies and percentages. Continuous variables were expressed as either mean ± standard deviation (SD) or median with interquartile range (IQR), depending on the distribution of the data.

Differences in the prevalence of virulence phenotypes among *Candida* species were assessed using Fisher–Freeman–Halton exact test with Monte Carlo simulation as appropriate. Comparisons involving more than two groups were performed using the Kruskal–Wallis test followed by Dunn’s post hoc test with Benjamini–Hochberg correction for multiple comparisons.

A two-sided *p*-value < 0.05 was considered statistically significant.

To evaluate pairwise relationships between fungal virulence traits (biofilm index and enzymatic activity scores) and antifungal susceptibility (MIC values), Spearman’s rank correlation analysis was performed. False discovery rate (FDR) was controlled across each 16-test correlation matrix using the Benjamini–Hochberg procedure. Due to intrinsic resistance, *Candida krusei* isolates were excluded from fluconazole-related analyses. Furthermore, a sensitivity analysis was restricted to the *Candida albicans* subcohort (*n* = 74) to verify that observed correlations were not driven by interspecies divergence. As a secondary analytical framework, univariate phenotyping and bivariate correlations were established as baseline profiles, while downstream supervised Random Forest classification and SHAP analyses were applied to probe whether these emergent phenotypic architectures align with or diverge from conventional taxonomic species boundaries.

#### 2.7.1. Feature Engineering for Multivariate Analysis

For multivariate analyses, enzymatic activity indices (Pz, Hz, and Prz), biofilm formation (biofilm index), and the minimum inhibitory concentrations (MICs) of antifungal agents were included as input features.

To integrate heterogeneous microbiological assays onto a common continuous scale ranging from 0 to 1, all variables were standardized. Because the original phospholipase (Pz), proteinase (Prz), and hemolysis (Hz) indices are inversely related to enzymatic activity, they were linearly inverted (formula 5) such that 0 denotes an absence of activity and values approaching 1.0 represent maximal enzymatic potency. The continuous biofilm index was rescaled to the [0, 1] interval via Min–Max normalization.

Transformed score = 1 − index(5)

An integrated virulence-related score (IVS) was calculated as unweighted arithmetic mean of the four normalized virulence-related features:

(6) $$IVS=\frac{Pzscore+Hzscore+Przscore+Bnorm}{4}$$

In clinical and experimental mycology, there is currently no validated consensus regarding the relative hierarchical contribution of individual virulence traits to mucosal colonization and tissue invasion. Therefore, an equal-weighting scheme was applied, with each of the four components contributing equally (weight = 0.25). Crucially, this metric does not represent an established qualitative clinical classification, nor does it quantify clinical pathogenicity in patients. Instead, it serves strictly as a laboratory-based surrogate measure of the overall in vitro virulence-related phenotypic burden per isolate, without imposing arbitrary investigator-defined assumptions.

To investigate the multidimensional architecture of the phenotypic data, a structured, three-tiered analytical workflow was implemented to address complementary biological questions:

First, principal component analysis (PCA) was performed to evaluate global linear variance across the standardized phenotypic traits and to determine whether overall pathogenic heterogeneity aligns with taxonomic species boundaries. The first two principal components were used to assess the extent of linear species-level segregation.

Second, because biological interactions among virulence factors and antifungal responses frequently involve complex nonlinear relationships, Uniform Manifold Approximation and Projection (UMAP) was applied strictly as an exploratory qualitative visualization tool. UMAP embeddings were generated using the Euclidean distance metric on standardized variables to examine local topological neighborhoods and potential phenotypic continua without imposing linear assumptions. Visual patterns in the 2D embedding were treated strictly as hypothesis-generating and were not used as standalone evidence of biological divergence.

Third, to objectively identify phenotypic subgroups without relying on low-dimensional visual projections, unsupervised agglomerative hierarchical clustering was performed directly on the full standardized multidimensional feature space. Cluster stability was assessed by bootstrap resampling with repeated estimation of the silhouette coefficient. To provide formal statistical verification, permutational multivariate analysis of variance (PERMANOVA) based on Euclidean distance matrices with 9999 permutations was executed, testing whether the identified phenotypic clusters represented statistically significant entities in multidimensional space.

Finally, the relative biological relevance and discriminatory importance of individual features were evaluated separately using downstream Random Forest and SHAP analyses, providing objective, data-driven insights into feature contributions.

#### 2.7.2. Machine Learning for Candida Species Classification

To evaluate the ability of phenotypic characteristics to discriminate among *Candida* species, a Random Forest classifier was developed. The target variable was species identity (*C. albicans*, *C. krusei*, *C. parapsilosis*, or *C. tropicalis*).

The predictor set included normalized phospholipase, proteinase, and hemolytic activity scores, the normalized biofilm index, and the minimum inhibitory concentrations (MICs) of fluconazole, voriconazole, itraconazole, and amphotericin B. The integrated virulence-related score and the phenotypic clusters derived from it were excluded from the model to avoid information leakage and preserve feature independence.

The Random Forest classifier was trained using 1000 decision trees with the Gini impurity criterion. To account for class imbalance, class weights were automatically adjusted in inverse proportion to class frequencies (class weight parameter set to “balanced”). Reproducibility was ensured by setting a fixed random state (random_state = 42). The minimum number of samples required at a terminal node was set to two, and the number of features considered at each split was defined as the square root of the total number of predictors.

Model performance was primarily evaluated using repeated stratified cross-validation (5 folds, 20 repeats; 100 total iterations) to account for sample variance and rare-class sensitivity. Aggregated out-of-fold predictions and empirical 95% confidence intervals (2.5th and 97.5th percentiles) were computed across iterations.

Given the imbalanced distribution of species, balanced accuracy and the macro-averaged F1-score were selected as the primary performance metrics. Overall accuracy, weighted F1-score, and class-specific precision, recall, and F1-score were also calculated. Model performance was compared with that of a baseline classifier assigning all observations to the most prevalent species.

The statistical significance of the classification performance was assessed by permutation testing using balanced accuracy as the primary metric and 1000 random permutations of the target labels. The complete cross-validation procedure was repeated for each permutation. Feature importance was evaluated using permutation importance and SHAP (SHapley Additive exPlanations) analysis.

### 2.8. Use of Generative Artificial Intelligence

During the preparation of this manuscript, the authors used GPT–5.6 Sol (OpenAI, San-Francisco, CA, USA) to assist with the refinement of statistical analysis code, English-language editing, and improvement of the clarity and structure of the manuscript. The tool was not used to generate, modify, or fabricate experimental data or to make autonomous scientific conclusions. All AI-assisted outputs were critically reviewed and verified by the authors, who take full responsibility for the accuracy, integrity, and final content of the manuscript.

## 3. Results

### 3.1. Characteristics of the Candida Isolates

A total of 186 participants were enrolled in the study, from whom 110 clinical *Candida* isolates were recovered and deposited in the culture collection. Mixed *Candida* colonization was identified in eight participants, including four cases of *C. albicans* + *C. krusei*, two cases of *C. albicans* + *C. tropicalis*, and two cases of *C. albicans* + *C. parapsilosis*. Co-colonizing isolates were treated as discrete observations in primary analyses because they represented phylogenetically distant species with distinct genomic backgrounds, although they shared identical host microenvironments.

Following prolonged cryopreservation at −80 °C, 107 isolates were successfully resuscitated and included in the subsequent analyses. Three isolates failed to revive due to a loss of biological viability during storage and were therefore excluded.

Among the recovered isolates, *C. albicans* was the predominant species, accounting for 69.2% (74/107) of all isolates (Table 1). Non-*albicans Candida* (NAC) species comprised the remaining 30.8% (33/107). Among the NAC isolates, *C. krusei* was the most frequently identified species (16.8%, 18/107), followed by *C. tropicalis* (7.5%, 8/107) and *C. parapsilosis* (6.5%, 7/107).

### 3.2. Biofilm Production of Candida Isolates

Among the 107 isolates examined, 58 (54.2%) were classified as non-biofilm producers, 36 (33.6%) as weak biofilm producers, and 13 (12.1%) as moderate biofilm producers. No isolates were classified as strong biofilm producers (Table 2).

Among *C. albicans* isolates, 55.4% were classified as non-biofilm producers, whereas 32.4% and 12.2% exhibited weak and moderate biofilm-forming capacity, respectively. Similarly, most *C. krusei* isolates (77.8%) were classified as non-biofilm producers. In contrast, the highest prevalence of biofilm-forming isolates was observed among *C. parapsilosis* and *C. tropicalis*. Among *C. parapsilosis* isolates, 71.4% were weak biofilm producers, whereas among *C. tropicalis* isolates, 50.0% and 25.0% were classified as weak and moderate biofilm producers, respectively.

Despite the observed differences in the distribution of biofilm-forming categories among *Candida* species, no statistically significant association was found between species and biofilm-forming capacity (Fisher–Freeman–Halton exact test with Monte Carlo simulation, *p* = 0.0577). Quantitative analysis of the biofilm index revealed significant overall variation among *Candida* species (Figure 1; Kruskal–Wallis test, H = 7.89, *p* = 0.048). While *C. parapsilosis* exhibited a nominally higher median biofilm index (1.229) compared to *C. krusei* (0.752), pairwise comparisons using Dunn’s test with Holm correction identified no statistically significant differences between individual species (all adjusted *p* > 0.05; strongest trend for *C. parapsilosis* vs. *C. krusei*, adjusted *p* = 0.060). Given the limited isolate count (*n* = 7), this elevated median should be regarded strictly as an unconfirmed exploratory trend.

### 3.3. Antifungal Susceptibility Profiles of Candida Isolates

Overall, the antifungal susceptibility profile of the clinical *Candida* isolates was characterized by the lowest minimum inhibitory concentration (MIC) values for voriconazole (MIC_50_ = 0.06 µg/mL; MIC_90_ = 0.125 µg/mL) and amphotericin B (MIC_50_ = 0.5 µg/mL; MIC_90_ = 1 µg/mL). Fluconazole exhibited identical MIC_50_ and MIC_90_ values of 2 µg/mL, whereas itraconazole demonstrated the highest overall values (MIC_50_ = 1 µg/mL; MIC_90_ = 4 µg/mL). Species-specific antifungal susceptibility is presented in Table 3.

Based on CLSI M60 clinical breakpoints, high susceptibility to fluconazole and voriconazole was observed across the cohort. Specifically, 97.2% (*n* = 72) of *C. albicans* isolates were susceptible to fluconazole, and 87.8% (*n* = 65) were susceptible to voriconazole, with the remainder displaying susceptible dose-dependent (SDD) profiles; no resistant isolates were detected. For *C. tropicalis*, susceptibility was 75.0% (*n* = 6) to fluconazole and 87.5% (*n* = 7) to voriconazole, with remaining isolates classified as SDD. All *C. parapsilosis* isolates (100%, *n* = 7) were fully susceptible to both fluconazole and voriconazole. *C. krusei* was considered intrinsically resistant to fluconazole; however, all 18 isolates exhibited voriconazole MICs ≤ 0.125 µg/mL well within the susceptible category (≤0.5 µg/mL).

According to CLSI M59 epidemiological cutoff values (ECVs), all 107 isolates (100%) across all four species exhibited amphotericin B MIC values (≤1 µg/mL) below the consensus threshold (≤2 µg/mL), confirming that all isolates belonged to the wild-type population devoid of acquired polyene mutational resistance (categorized descriptively as susceptible in Table 3). For itraconazole, categorical clinical classifications (S/SDD/R) were omitted due to the absence of established CLSI CBPs; across species, 100% of *C. parapsilosis* and *C. tropicalis* isolates fell within the WT category (MIC ≤ 0.5 µg/mL), whereas *C. krusei* exhibited non-wild-type MIC levels (MIC_50/90_ = 4 µg/mL, above the 1 µg/mL ECV), and *C. albicans* spanned both WT and NWT distributions (range 0.5–4 µg/mL} against an ECV of 0.125 µg/mL).

Nonparametric analysis identified a statistically significant interspecies difference in continuous MIC distributions solely for itraconazole (Kruskal–Wallis test, H = 59.47, *p* < 0.001), driven by elevated MICs in *C. krusei*. In contrast, no significant interspecies differences were observed for voriconazole (H = 1.30, *p* = 0.729), amphotericin B (H = 1.16, *p* = 0.763), or fluconazole (H = 6.31, *p* = 0.097).

### 3.4. Enzymatic Virulence Factors Among Candida Species

To assess species-specific differences in enzymatic virulence, the prevalence of phospholipase, hemolytic, and proteinase activities was compared among the identified *Candida* species (Table 4).

Evaluation of hydrolytic enzyme production revealed that hemolytic activity was the most prevalent virulence trait across the cohort, detected in approximately half of the isolates across all taxa (50.0–57.1%; Table 4). Phospholipase and proteinase activities were observed in smaller subsets across the four species (25.0–57.1% and 25.7–42.9%, respectively; Table 4).

Nonparametric analysis demonstrated that enzymatic virulence profiles did not differ significantly among the evaluated *Candida* species, with comparable activity distributions observed for phospholipase (Kruskal–Wallis H = 3.45, *p* = 0.327), hemolytic activity (H = 1.16, *p* = 0.762), and proteinase (H = 1.67, *p* = 0.643; Table 4). Overall, hydrolytic enzyme secretion was characterized by pronounced intraspecies heterogeneity without statistically significant species-specific partitioning.

### 3.5. Association Between Virulence-Related Phenotypes and Antifungal Susceptibility

Spearman correlation analysis revealed predominantly weak associations between virulence-related phenotypic traits and antifungal MIC values. In the pooled analysis, phospholipase activity showed a weak inverse correlation with fluconazole MIC (ρ = −0.222, *p* = 0.0369), while biofilm formation was weakly inversely correlated with itraconazole MIC (ρ = −0.195, *p* = 0.0443). However, neither association remained statistically significant after Benjamini–Hochberg correction for multiple comparisons (FDR-adjusted *p* = 0.3541 for both). No significant correlations were observed between proteinase or hemolytic activity and MIC values for the tested antifungal agents (Figure 2).

A sensitivity analysis restricted to *C. albicans* showed a similar pattern. Biofilm formation was weakly inversely correlated with fluconazole MIC (ρ = −0.286, *p* = 0.0136), and phospholipase activity showed a weak inverse correlation with fluconazole MIC (ρ = −0.233, *p* = 0.0461). These associations also did not remain significant after FDR correction (adjusted *p* = 0.2176 and 0.3685, respectively). Overall, no virulence-related phenotypic trait demonstrated a statistically significant association with antifungal MIC values after correction for multiple testing.

### 3.6. Integrated Virulence-Related Score (IVS) of Clinical Candida Isolates

Across all 107 clinical isolates, the median integrated virulence-related score was 0.141 (IQR 0.100–0.239), with a mean value of 0.167 ± 0.090. The highest median IVS was observed in *C. parapsilosis* (0.228; IQR 0.197–0.243), followed by *C. tropicalis* (0.176; IQR 0.148–0.204), *C. krusei* (0.136; IQR 0.098–0.202), and *C. albicans* (0.129; IQR 0.086–0.241).

Although *C. parapsilosis* and *C. tropicalis* exhibited higher median IVS values, this observation is based on a limited sample size (*C. parapsilosis*, *n* = 7; *C. tropicalis*, *n* = 8) and the overall differences among species were not statistically significant (Kruskal–Wallis test, H = 4.868, *p* = 0.182). The marked overlap in index distributions across species suggests substantial intraspecies variability in phenotypic pathogenic potential (Figure 3).

### 3.7. Multivariate Analysis of Phenotypic Virulence and Machine Learning

Because conventional univariate analyses and bivariate correlations demonstrated that individual virulence attributes varied largely independently of species affiliation and antifungal MICs, a structured, tiered multivariate workflow was implemented to resolve the central objective of this study: uncovering latent phenotypic clusters. Within this framework, principal component analysis (PCA) and Uniform Manifold Approximation and Projection (UMAP) served strictly as complementary linear and nonlinear exploratory tools to visualize cohort dispersion. The primary endpoint—unsupervised agglomerative hierarchical clustering validated by PERMANOVA—was executed directly on the full standardized multidimensional matrix to objectively delineate species-independent pathogenic entities. Finally, supervised Random Forest modeling and SHAP interpretability were utilized as downstream validation instruments to evaluate the extent to which these multidimensional phenotypes could predict or differentiate taxonomic species.

#### 3.7.1. Principal Component Analysis of Phenotypic Virulence Traits

To explore the multidimensional phenotypic relationships among the clinical *Candida* isolates, principal component analysis (PCA) was performed. The first two principal components explained 34.9% of the total variance and captured the dominant patterns of phenotypic variation. Species formed partially overlapping distributions without clear taxonomic separation (Figure 4). For visualization purposes, isolates were additionally colored according to the previously calculated IVS and classified into low-, moderate-, and high-risk phenotypic groups. Although species separation remained incomplete, isolates tended to group according to phenotypic risk categories. High-risk isolates were distributed toward the negative values of PC1 and positive values of PC2, whereas low-risk isolates clustered closer to the center and positive PC1 values, indicating that overall phenotypic pathogenicity contributed more strongly to the principal component structure than species identity alone.

The PCA score plot demonstrated substantial overlap among *C. albicans*, *C. krusei*, *C. tropicalis*, and *C. parapsilosis* isolates. Isolates of different species occupied overlapping regions of the multidimensional phenotypic space and did not form distinct species-specific clusters. These findings indicate that the investigated phenotypic characteristics could not be explained solely by species identity. Nevertheless, a tendency toward partial segregation according to phenotypic risk categories was observed, suggesting that the overall combination of phenotypic traits contributed more strongly to the multidimensional structure than species identity alone.

#### 3.7.2. UMAP Visualization of Multidimensional Phenotypic Relationships

To visually inspect potential nonlinear continuous relationships among the multi-assay phenotypes, Uniform Manifold Approximation and Projection (UMAP) was applied as an exploratory visualization tool. The resulting 2D embedding displayed broad phenotypic gradients across the clinical cohort (Figure 5). Consistent with the PCA findings, isolates belonging to different *Candida* species showed marked overlap across the manifold, confirming the absence of strict species-bound phenotypic separation. High-risk isolates exhibited local proximity along the embedding continuum, whereas low- and moderate-risk isolates were distributed across adjoining regions. However, because 2D distance metrics in nonlinear manifold learning do not represent formal statistical proof of distinct groups, these visual patterns were interpreted strictly as exploratory, with formal cluster validation delegated to subsequent non-parametric distance-based testing (Figure 5).

#### 3.7.3. Hierarchical Clustering Identifies Phenotypic Pathogenicity Profiles

To identify objective phenotypic groups independently of visual 2D projections, unsupervised agglomerative hierarchical clustering was executed directly on the full standardized feature matrix (comprising enzymatic activities, biofilm index, and antifungal MICs). The analysis identified three phenotypic clusters, hereafter referred to as Phenotypic Clusters I–III.

Projection of the clustering results onto the UMAP embedding demonstrated partial spatial separation of the three clusters, although their boundaries remained continuous rather than discrete (Figure 6). Phenotypic Cluster II (*n* = 13) formed the most compact region of the embedding, whereas Phenotypic Cluster III (*n* = 31) occupied a distinct neighboring region. In contrast, Phenotypic Cluster I (*n* = 63) showed the greatest within-cluster dispersion, indicating higher phenotypic heterogeneity among its constituent isolates. To determine whether the observed clustering represented a statistically significant phenotypic structure, the cluster assignments were subsequently evaluated using PERMANOVA.

To facilitate the biological interpretation of the identified phenotypic clusters, enzymatic activities, biofilm-forming capacity, and antifungal susceptibility profiles were compared across the three clusters (Table 5).

Analysis of the phenotypic characteristics demonstrated significant differences among the three phenotypic clusters in phospholipase activity (Kruskal–Wallis test, H = 95.73, *p* < 0.001) and the IVS (H = 42.11, *p* < 0.001). In contrast, no significant differences were observed in itraconazole MIC values (H = 2.86, *p* = 0.240), proteinase activity, hemolytic activity, or biofilm-forming capacity (all *p* > 0.05).

Pairwise comparisons using Dunn’s test with Holm correction confirmed that Phenotypic Cluster III exhibited significantly higher phospholipase activity than Clusters I and II (both *p* < 0.001; Table 6). Consequently, the integrated virulence-related phenotype was also significantly elevated in Cluster III (*p* < 0.001 vs. Cluster I; *p* = 0.005 vs. Cluster II).

Crucially, this difference in the IVS must not be interpreted as an independent biological validation of Cluster III. Because this composite metric is derived directly from the underlying virulence attributes (specifically incorporating the normalized phospholipase score), its elevation is largely an arithmetic reflection of marked phospholipase secretion. Among the four primary virulence traits evaluated, phospholipase activity represented the sole biological driver distinguishing Cluster III from Clusters I and II, while proteinase, hemolysis, and biofilm formation remained comparable across all three groups (Table 7). Therefore, the IVS serves strictly as an internal descriptive summary of cumulative pathogenic burden.

The species composition did not differ significantly among the identified clusters (*p* = 0.648). Each cluster comprised representatives of multiple *Candida* species (Table 8).

Isolates of *C. albicans*, *C. krusei*, and *C. tropicalis* were present in all three clusters, whereas *C. parapsilosis* was absent only from Cluster II. These findings indicate similarities in multidimensional phenotypic characteristics.

To evaluate the statistical robustness of the clustering results, a permutational multivariate analysis of variance (PERMANOVA) was performed. Significant differences were identified among the multidimensional phenotypic profiles of the three clusters (pseudo-F = 6.72, *p* = 0.0002). These findings confirm that the identified clusters represent genuine differences in multidimensional space rather than artifacts arising from the UMAP visualization.

#### 3.7.4. Machine Learning Classification and Feature Importance Analysis

A Random Forest classifier trained on virulence-associated phenotypes and antifungal susceptibility profiles achieved an aggregated out-of-fold accuracy of 0.785 and a mean accuracy of 0.793 ± 0.017 (95% Empirical interval: 0.757–0.818) across 20 repeated 5-fold cross-validation runs, outperforming the majority-class baseline (0.692). Across repeated cross-validation iterations, the model maintained stable performance above the baseline, achieving a mean balanced accuracy of 0.518 ± 0.030 (95% Empirical interval: 0.461–0.577 vs. 0.250 baseline) and a macro-F1 score of 0.504 ± 0.031 (95% Empirical interval: 0.444–0.559 vs. 0.204 baseline) (Table 9).

The Random Forest model consistently outperformed the majority-class baseline classifier across all evaluated performance metrics. Classification performance was highest for *C. albicans* and *C. krusei*, whereas none of the *C. tropicalis* isolates were correctly identified (Figure 7, Table 10).

A detailed breakdown of the consensus out-of-fold predictions reveals that classification performance remained robust for the most prevalent species: 17 of 18 *C. krusei* isolates were correctly identified (sensitivity: 0.944, precision: 0.810, F1-score: 0.872), and 65 of 74 *C. albicans* isolates were accurately classified (sensitivity: 0.878, precision: 0.855, F1-score: 0.867; Table 10, Figure 7). Conversely, the classifier exhibited limited discriminative capacity for taxa with low sample prevalence. Only two of seven *C. parapsilosis* isolates were correctly predicted (sensitivity: 0.286, precision: 0.286, F1-score: 0.286), while five were misclassified as *C. albicans*. Furthermore, all eight *C. tropicalis* isolates were misclassified (six as *C. albicans* and two as *C. krusei*), yielding a recall and F1-score of 0.000.

Overall, these findings indicate that the evaluated phenotypic traits harbor distinctive signatures capable of separating *C. albicans* and *C. krusei*. The constrained predictive accuracy for *C. tropicalis* and *C. parapsilosis* reflects both their limited numerical representation in the training cohort and broad phenotypic overlap with *C. albicans*.

#### 3.7.5. SHAP-Based Model Interpretation

To assess the contribution of individual phenotypic characteristics to the multiclass classification of *Candida* species, the Random Forest model was interpreted using SHAP (SHapley Additive exPlanations) analysis (Figure 8). Global feature importance analysis identified itraconazole MIC as the dominant predictor, yielding the highest mean absolute SHAP value (0.1110). Among the virulence-associated characteristics, biofilm formation was the most informative feature (0.0427). Smaller contributions were observed for phospholipase activity (0.0267), hemolytic activity (0.0204), and proteinase activity (0.0171).

These findings are consistent with the antifungal susceptibility analysis, in which itraconazole MIC was the only susceptibility parameter that differed significantly among *Candida* species. In contrast, amphotericin B, fluconazole, and voriconazole MIC values showed comparatively minor contributions to species classification, with mean absolute SHAP values of 0.0100, 0.0074, and 0.0033, respectively. Overall, itraconazole MIC and biofilm formation served as the strongest predictive features distinguishing the species profiles, reflecting characteristic phenotypic associations rather than mechanistic causal drivers.

To further characterize the direction and magnitude of feature effects, class-specific SHAP analysis was performed separately for each *Candida* species (Figure 9).

For *C. albicans*, itraconazole MIC was the most influential feature (0.1525). Lower itraconazole MIC values shifted the model predictions toward the *C. albicans* class, whereas higher values reduced this probability. Among the virulence-associated characteristics, biofilm formation (0.0518) and phospholipase activity (0.0474) made the greatest contributions, with higher values for both increasing the likelihood of classification as *C. albicans*.

For *C. krusei*, itraconazole MIC was likewise the dominant predictor (0.1820). However, the effect was inverted: higher itraconazole MIC values strongly shifted predictions toward the *C. krusei* class. Biofilm formation contributed less (0.0329), while the remaining phenotypic characteristics had only negligible effects on species classification. The prominent role of itraconazole MIC aligns with the high classification performance achieved for *C. krusei*.

Classification of *C. parapsilosis* relied on a more balanced combination of phenotypic characteristics. The largest contributions were provided by itraconazole MIC (0.0667), biofilm formation (0.0443), and phospholipase activity (0.0348). Higher biofilm-forming capacity and phospholipase activity increased the probability of predicting *C. parapsilosis.* Proteinase (0.0200) and hemolytic (0.0185) activities showed comparatively small but measurable contributions to species discrimination.

For *C. tropicalis*, all investigated characteristics exhibited low SHAP values. The largest contributions were observed for itraconazole MIC (0.0429) and biofilm formation (0.0418); however, the corresponding SHAP values were concentrated close to zero, indicating a lack of distinct phenotypic features capable of reliably distinguishing *C. tropicalis* from the other species. This observation is consistent with the poor classification performance of the model and the failure to correctly identify any *C. tropicalis* isolates.

Overall, the SHAP analysis demonstrated that the diagnostic importance of individual phenotypic features differed among *Candida* species. Itraconazole MIC represented the principal discriminative feature for *C. albicans* and *C. krusei*, whereas biofilm formation and phospholipase activity provided additional discriminatory information for *C. albicans* and *C. parapsilosis*. In contrast, the investigated phenotypic profile did not provide sufficient discriminatory power to reliably distinguish *C. tropicalis* from the remaining species.

Collectively, these findings indicate that Random Forest classification of *Candida* species relied not on a single universal marker, but rather on species-specific combinations of itraconazole susceptibility and virulence-associated traits.

Taken together, the results of PCA, UMAP, hierarchical clustering, PERMANOVA, Random Forest classification, and SHAP analysis demonstrate that the phenotypic organization of the *Candida* population only partially aligns with conventional species classification. Despite substantial overlap among species-specific phenotypic profiles, multidimensional analyses identified a statistically significant internal phenotypic structure. Among the investigated species, *C. krusei* exhibited the clearest phenotypic differentiation, whereas *C. tropicalis* showed the greatest overlap with other species, explaining its poor classification performance.

## 4. Discussion

The principal contribution of the present study is the identification and statistical validation of three discrete multidimensional phenotypic virulence clusters among clinical *Candida* isolates using unsupervised machine learning. Notably, these phenotypic clusters transcended conventional taxonomic species boundaries, demonstrating that pathogenic potential is governed by modular combinations of virulence attributes. In this workflow, single-factor phenotypic assays and the composite virulence score served as foundational baseline measures, while supervised classification affirmed that conventional species designations fail to capture the emergent multidimensional organization of clinical isolates.

These findings should be interpreted in the context of the COVID-19 pandemic, during which the widespread use of broad-spectrum antibiotics, systemic glucocorticoids, prolonged hospitalization, and SARS-CoV-2-associated immune dysregulation may have created favorable conditions for *Candida* colonization of mucosal surfaces [37,38]. Furthermore, alterations in the microbiota and impairment of epithelial barrier integrity associated with SARS-CoV-2 infection may have modified the ecological niche of mucosal surfaces, thereby facilitating the persistence and dissemination of *Candida* strains with diverse phenotypic characteristics, including enhanced biofilm-forming capacity and the secretion of hydrolytic enzymes [39,40,41]. However, the absence of a comparison group comprising isolates collected before the pandemic or from individuals without COVID-19 precludes the direct attribution of the observed phenotypic heterogeneity to SARS-CoV-2 infection. Therefore, the present findings should be interpreted as describing the phenotypic profile of a *Candida* population circulating within a specific epidemiological and clinical context.

Although biofilm formation and itraconazole susceptibility differed significantly among *Candida* species, most of the remaining virulence-associated characteristics showed no significant species-specific variation. This indicates that the pathogenic potential of clinical isolates cannot be defined solely by taxonomic affiliation, as combinations of independent phenotypic traits play a crucial role. In particular, while *C. parapsilosis* displayed an exploratory trend toward a higher median IVS, this non-significant difference (*p* = 0.182) should not be overinterpreted as evidence of heightened pathogenic virulence, especially given the marked distributional overlap and the small sample size (*n* = 7). Consequently, evaluating individual features alone does not adequately capture the complexity of phenotypic diversity among clinical *Candida* isolates.

This premise is further corroborated by pairwise Spearman correlation analysis, which revealed no statistically significant monotonic associations between individual virulence traits and antifungal MICs following Benjamini–Hochberg FDR correction (all adjusted *p* > 0.05; Figure 2). The lack of robust covariation demonstrates that hydrolytic enzyme secretion, biofilm formation, and antifungal susceptibility segregate as functionally independent phenotypic axes rather than physiologically linked traits. Consequently, virulence trait expression and antifungal susceptibility cannot be reliably predicted from one another via bivariate models. Collectively, the absence of both strict taxonomic segregation and direct bivariate links underscores the limitations of univariate metrics, providing a compelling rationale for employing multivariate clustering and machine-learning frameworks to capture the emergent phenotypic profiles of clinical *Candida* isolates.

One of the principal findings of this study was the identification of three phenotypic clusters among clinical *Candida* isolates using unsupervised machine learning approaches. Each cluster comprised representatives of multiple *Candida* species, whereas isolates belonging to the same species were distributed across different phenotypic groups. These findings indicate that the phenotypic organization of pathogenic traits does not fully align with the taxonomic structure of the genus *Candida*. Rather, the identified clusters represent shared multidimensional phenotypic states that may emerge under similar ecological and host-related selective pressures, irrespective of species identity [3,42].

Pronounced intraspecies heterogeneity has previously been demonstrated among clinical isolates of *C. albicans*, which differ in their capacity for biofilm formation, adhesion, filamentation, and the production of other virulence factors, with these properties varying according to the morphological state of the cells [7]. Consistent with these observations, Gabaldón and Carreté proposed that fungal virulence is a complex evolutionary trait whose expression depends on environmental conditions and host-derived selective pressures [43]. They further emphasized that pathogenic potential cannot be reliably predicted from a single virulence-associated characteristic or from the simple additive effects of individual traits, as different fungal species may employ similar mechanisms of adaptation, persistence, and dissemination [43].

The application of unsupervised learning approaches appears justified for identifying latent phenotypic structures among clinical *Candida* isolates. Consistent with our findings, Beyer et al. (2025) identified three major phenotypic archetypes and several subclusters based on the quantitative assessment of adaptive and phenotypic characteristics [44]. Together, these studies suggest that multidimensional phenotypic organizations can reveal biologically meaningful patterns that are not readily apparent using conventional species-based analyses [44]. Notably, isolates of individual *Candida* species, including *C. albicans* and *C. tropicalis*, were distributed across multiple phenotypic clusters, whereas each cluster comprised representatives of several species, indicating substantial intra- and interspecies phenotypic heterogeneity.

Furthermore, Angiolini et al. suggested that the clinical manifestations of candidiasis are associated not with individual virulence-associated characteristics, but with their multidimensional combinations, including enzymatic activity, biofilm formation, adaptive properties, and antifungal susceptibility [45]. Whereas their study was restricted to a single species, the present findings demonstrate that multidimensional phenotypic organization extends across several clinically important *Candida* species, suggesting that phenotypic clustering is not limited to *C. albicans* but may represent a broader feature of the genus. Together with the results of the present study, these observations support the concept that integrated phenotypic analysis provides a more informative framework for characterizing the biological diversity and pathogenic potential of *Candida* than the evaluation of individual phenotypic traits in isolation.

The identified clusters represent a continuum of multidimensional phenotypic states rather than discrete categories of “low” or “high” virulence. Cluster I exhibited the lowest overall pathogenicity profile, Cluster II displayed an intermediate phenotype, and Cluster III was distinguished primarily by high phospholipase activity (accompanied by an arithmetically elevated composite IVS), while remaining comparable across other evaluated traits. However, because biofilm formation, hemolytic activity, and proteinase activity did not differ significantly among the clusters, it is evident that the enhancement of one virulence trait is not necessarily accompanied by proportional changes in others. This indicates that different pathogenic features may vary independently within clinical *Candida* isolates. Consequently, pathogenic potential emerges from distinct combinations of complementary traits rather than a uniformly increased expression of all virulence factors.

This interpretation aligns with accumulating evidence of pronounced phenotypic heterogeneity among clinical *Candida* isolates. Brandquist et al. (2023) reported substantial variation in the filamentation capacity of *C. albicans* under different inducing conditions, highlighting considerable variability in a key virulence trait even within a single species [46]. Similarly, Mroczyńska and Brillowska-Dąbrowska demonstrated significant variability in hydrolytic enzyme production, biofilm formation, and virulence in a *Galleria mellonella* model across clinical *Candida* isolates [11]. Collectively, these reports reinforce the conclusion that species identity alone fails to adequately explain the diversity of clinically relevant phenotypes. Instead, isolates of the same species may display distinct multidimensional profiles, whereas isolates from different taxonomic groups may share similar phenotypic states.

By integrating multiple characteristics into a unified multidimensional framework, machine learning expands the diagnostic and analytical utility of conventional microbiological methods, revealing complex patterns hidden from univariate analyses.

Given the functional decoupling of virulence attributes and the inherent limitations of conventional azoles against non-*ю* taxa such as *Candida krusei*, addressing persistent mucosal colonization requires therapeutic frameworks that extend beyond traditional monotherapies. In this regard, novel antimicrobial paradigms—including the repurposing of non-antifungal agents and the deployment of advanced nanotherapeutics—represent promising avenues to circumvent fungal resistance and attenuate multidimensional virulence phenotypes [47].

Several limitations of this study warrant consideration. First, all isolates were recovered from a single center (Almaty, Kazakhstan) and originated exclusively from COVID-19 convalescent patients without a non-COVID-19 or pre-pandemic control cohort. Consequently, these findings characterize a specific post-viral population, and their generalizability to broader clinical settings remains to be confirmed. Second, phenotypic characterization was restricted to selected enzymatic and biofilm assays without corresponding genomic profiling, leaving the underlying molecular mechanisms unaddressed. Additionally, biofilm formation was evaluated using technical triplicates derived from a single standardized culture per isolate; while appropriate for high-throughput screening of a 107-isolate clinical cohort, future studies should incorporate multi-day biological replicates to capture potential culture-to-culture physiological variation. Third, detailed clinical and pharmacological metadata—such as underlying host comorbidities, severity of viral infection, systemic corticosteroid dosing, and prior exposure to antifungal agents- were not systematically captured within the study registry. Consequently, the potential selective impact of prior antifungal pressure or specific host immune impairments on the observed MIC distributions and virulence phenotypes could not be directly adjusted for in multivariate models. Finally, mixed-species colonization was identified in eight participants. Although these isolates were analyzed as discrete observations due to distinct phylogenetic species boundaries, those recovered from the same host shared microenvironmental exposures and may be partially correlated; future multicenter studies should incorporate dedicated hierarchical modeling to account for this within-participant dependence.

The modest sample size (*n* = 107) and the limited representation of *C. tropicalis* (*n* = 8) and *C. parapsilosis* (*n* = 7) constrain the precision of the supervised modeling. Repeated cross-validation provided an internal assessment of classification performance, but it does not completely exclude the risk of overfitting or replace the need for external validation. SHAP values were deployed to describe feature contributions to model predictions and should be interpreted as predictive phenotypic correlates rather than causal biological drivers.

## 5. Conclusions

The present study demonstrates that the pathogenic potential of clinical *Candida* isolates is governed by multidimensional combinations of virulence traits. By applying unsupervised machine learning, we identified latent phenotypic clusters that span across multiple *Candida* species, capturing biological architectures that remain undetectable by conventional univariate analyses. Looking forward, integrating multidimensional phenotypic profiling into prospective clinical cohorts may provide a valuable framework to investigate whether specific in vitro virulence clusters can differentiate harmless mucosal colonizers from strains with invasive potential in vulnerable patients.

## Figures and Tables

**Figure 1:**
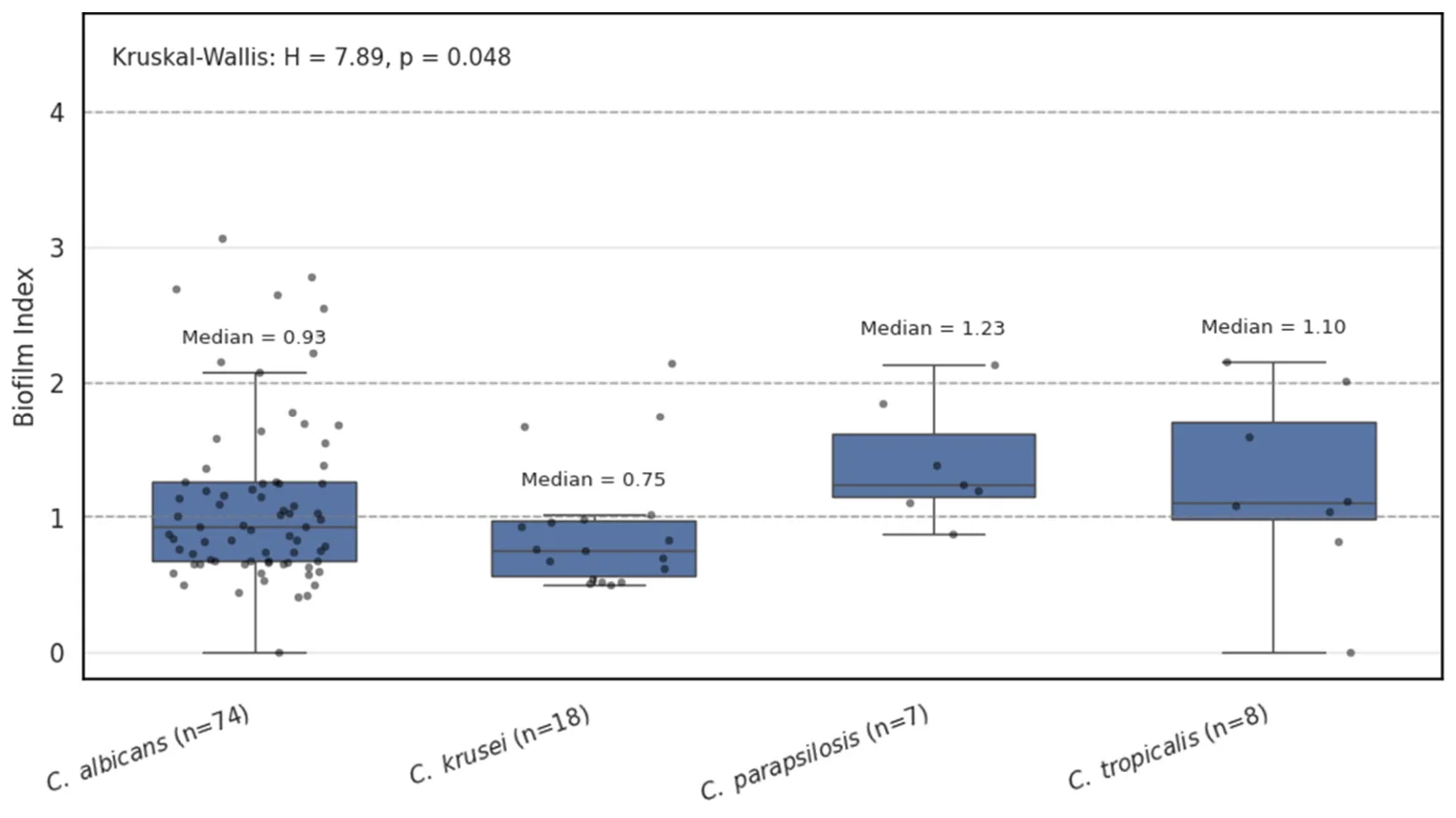
Distribution of the biofilm index across *Candida* species. Boxes represent the median and interquartile range, whiskers extend to 1.5 times the interquartile range, and individual points represent individual isolates.

**Figure 2:**
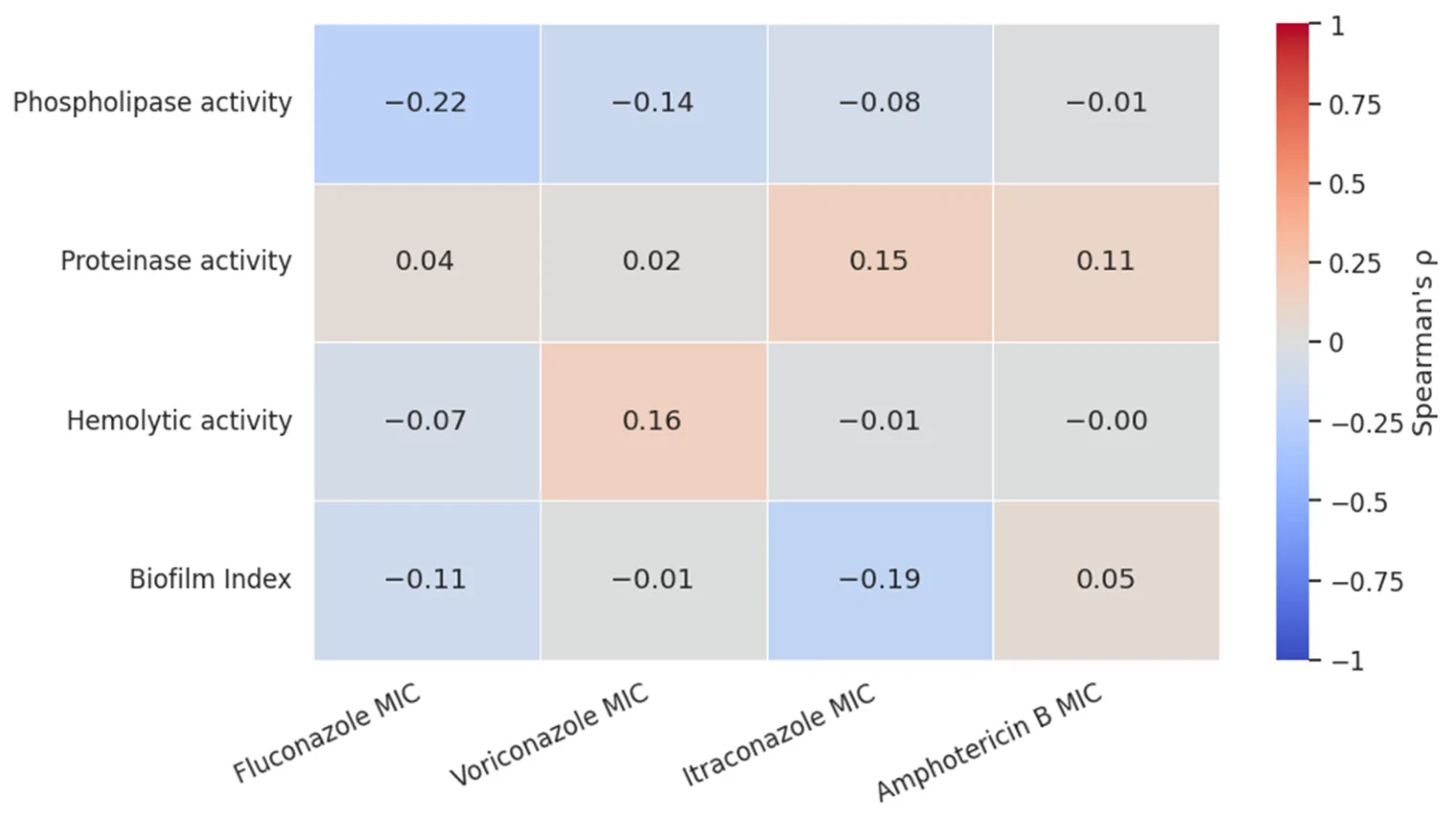
Correlations between virulence-related phenotypic traits and antifungal MIC values.

**Figure 3:**
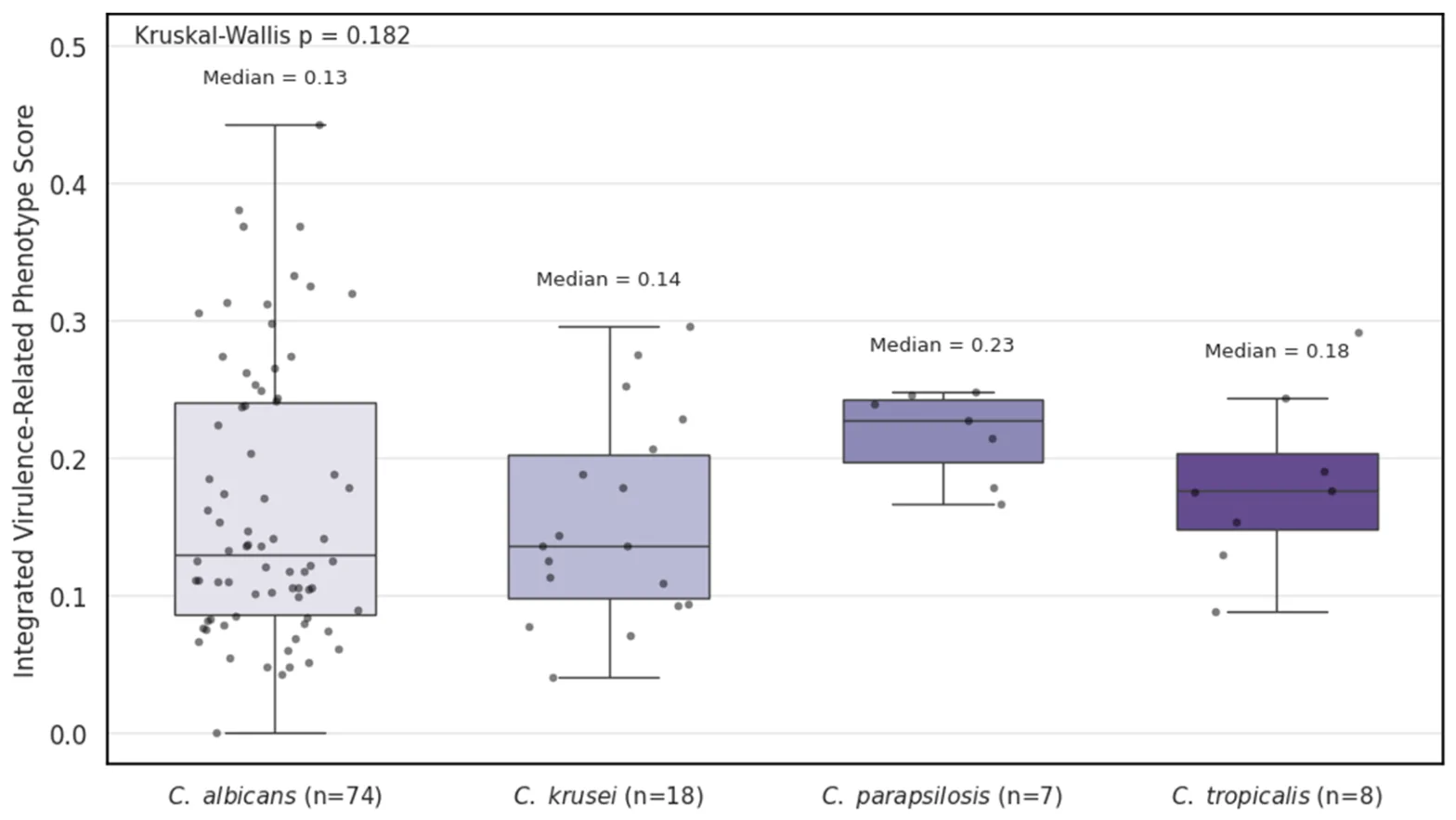
Distribution of the integrated virulence-related phenotype score among *Candida* spp. (Kruskal–Wallis H = 4.868, *p* = 0.182).

**Figure 4:**
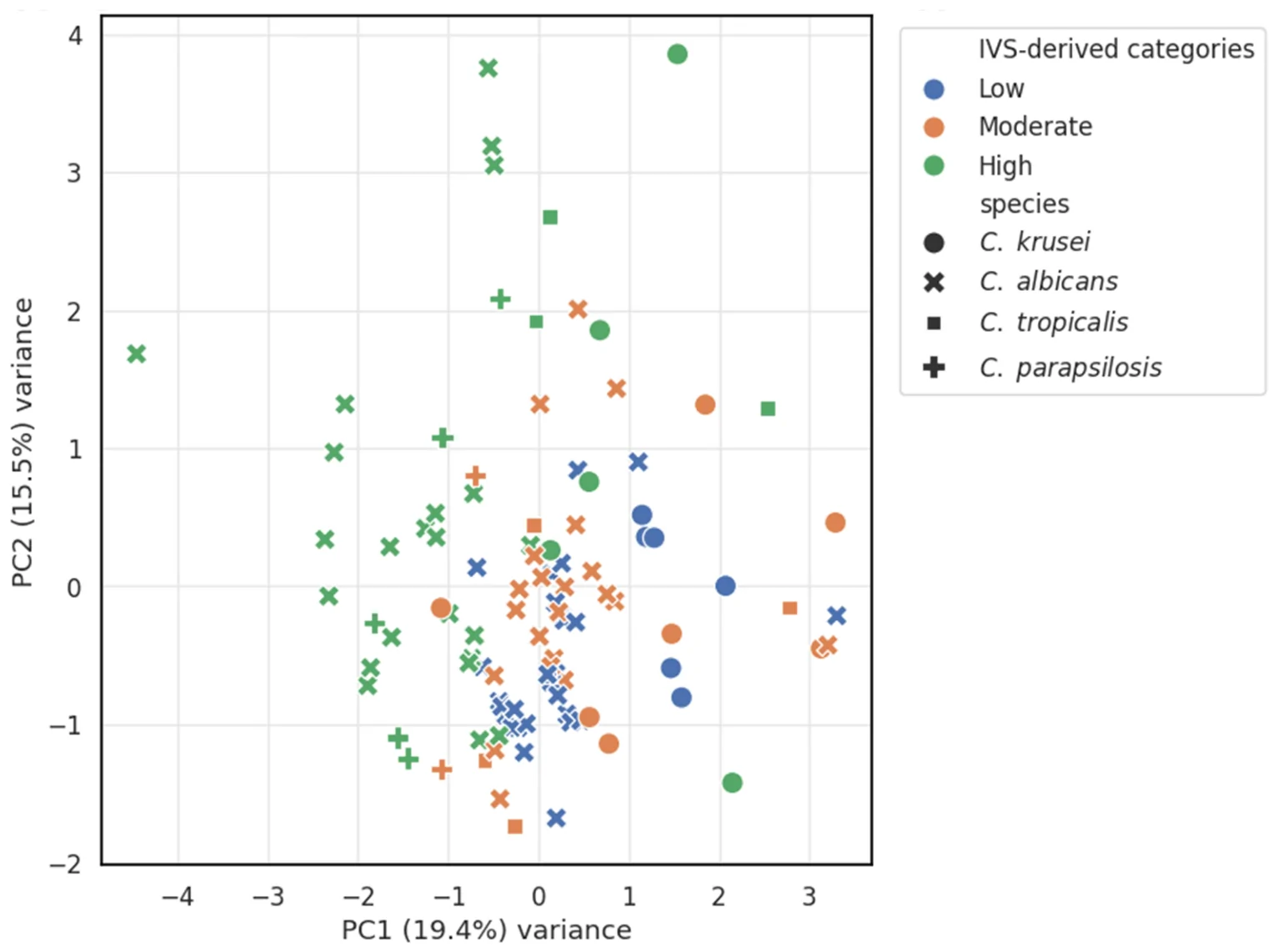
Principal component analysis (PCA) of multidimensional phenotypic profiles of clinical *Candida* isolates. Note: PCA was performed using phospholipase, proteinase, and hemolytic activities, biofilm-forming capacity, and antifungal susceptibility. Colors indicate phenotypic risk groups derived from the integrated virulence-related score, whereas symbols represent *Candida* species.

**Figure 5:**
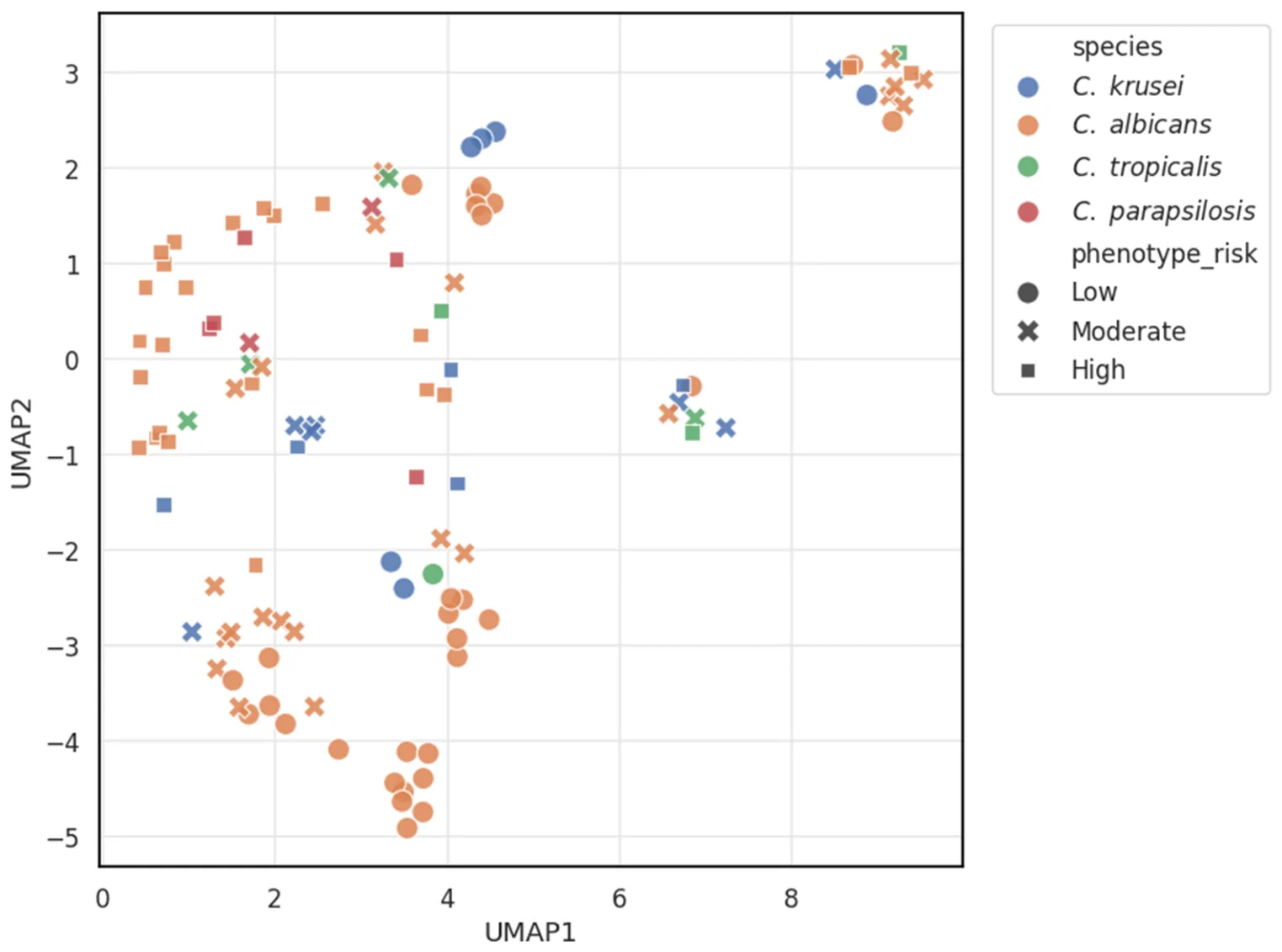
Two-dimensional UMAP of multidimensional phenotypic profiles of clinical *Candida* isolates. Note: Nearby points in the UMAP embedding represent isolates with similar multidimensional phenotypic profiles based on virulence traits, biofilm-forming capacity, and antifungal susceptibility. The UMAP1 and UMAP2 axes are arbitrary embedding coordinates and do not have an independent biological interpretation. Colors denote *Candida* species, whereas symbols indicate phenotypic risk categories.

**Figure 6:**
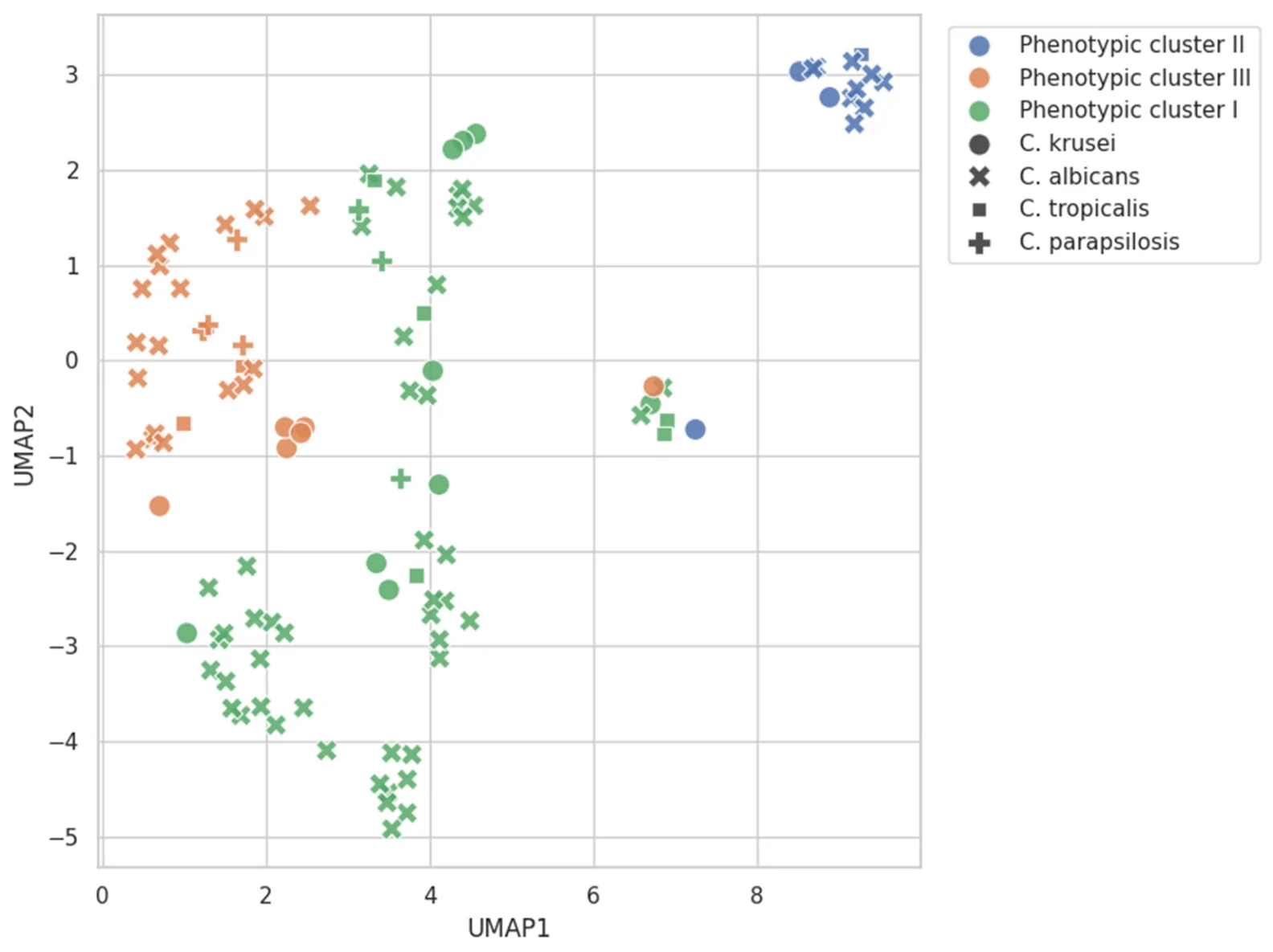
Visualization of phenotypic clusters of clinical *Candida* isolates in the UMAP embedding. Note: Three phenotypic clusters were identified by unsupervised hierarchical clustering based on virulence traits, biofilm-forming capacity, and antifungal susceptibility. The relative proximity of clusters in the UMAP embedding reflects similarity in the multidimensional phenotypic profiles of the clinical isolates.

**Figure 7:**
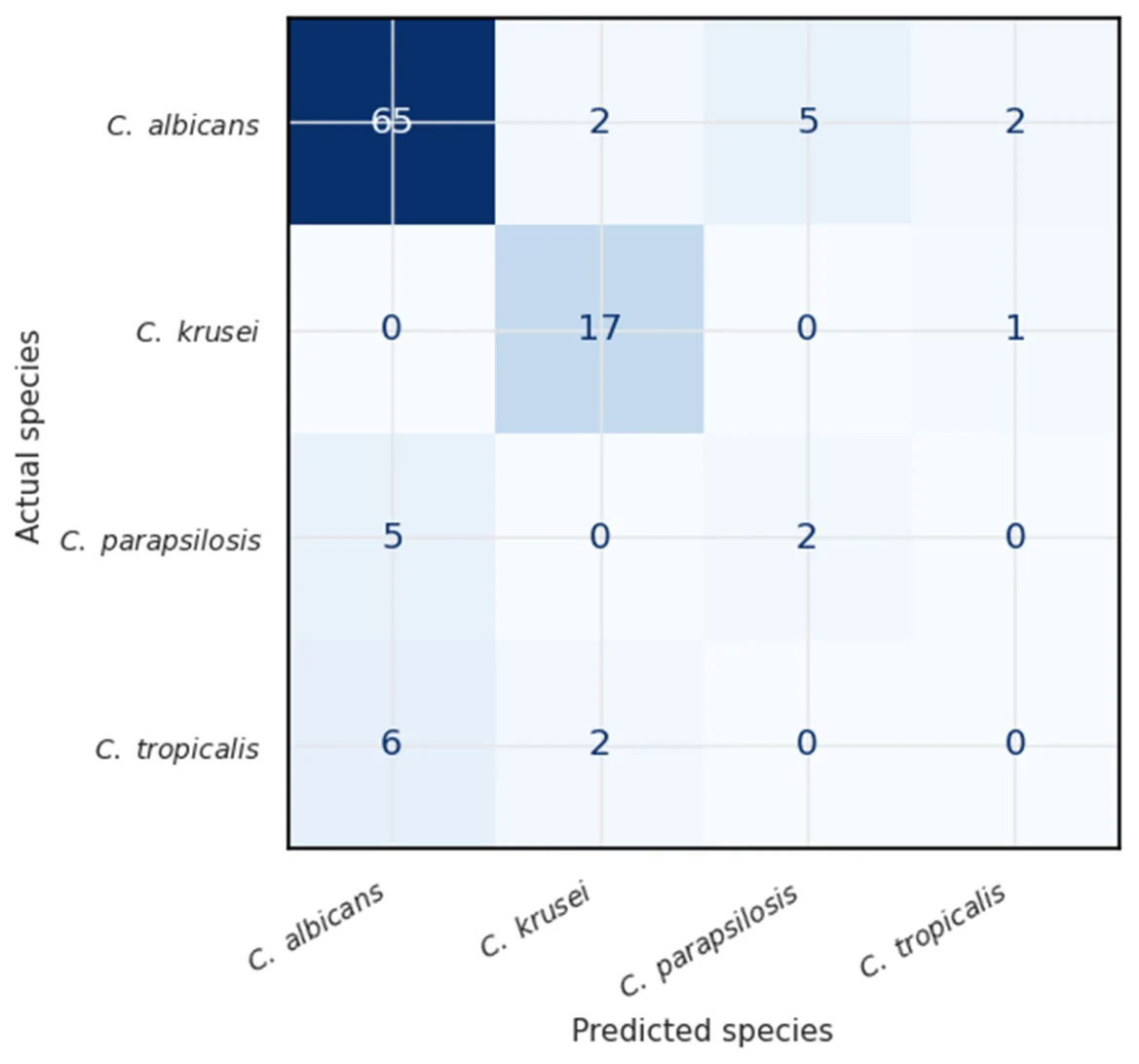
Confusion matrix of repeated cross-validated Random Forest classification of *Candida* species. The matrix was constructed using consensus out-of-fold predictions obtained from repeated stratified five-fold cross-validation performed over 20 repeats. Rows indicate the true species labels, whereas columns indicate the predicted species labels assigned by the Random Forest model. Each cell shows the number of isolates within each true-predicted species combination.

**Figure 8:**
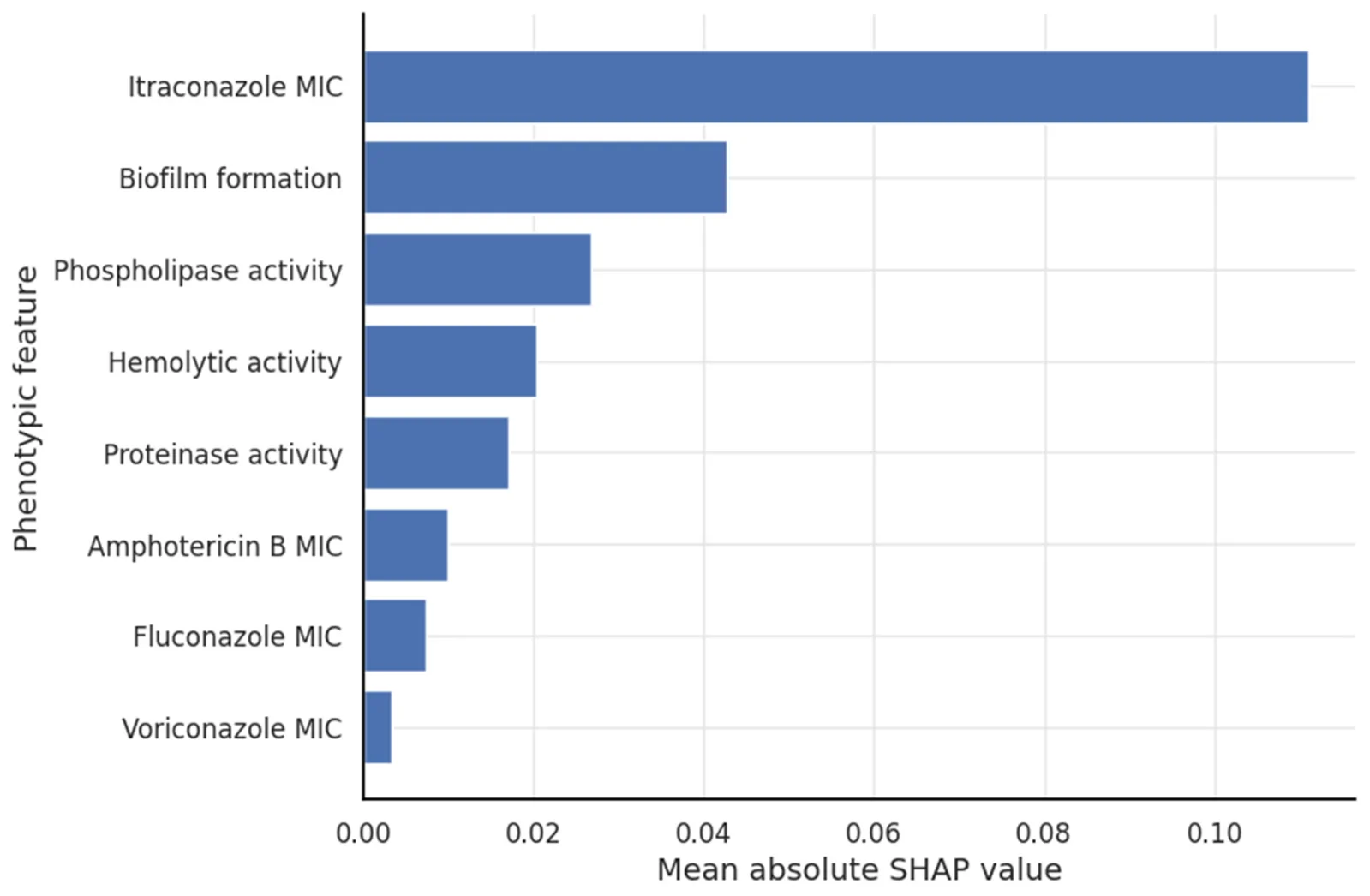
Global feature importance in the Random Forest model based on SHAP analysis. Mean absolute SHAP values are shown for each feature, averaged across all isolates and all four *Candida* species. Higher values indicate a greater overall contribution of the corresponding feature to species classification, irrespective of the direction of its effect.

**Figure 9:**
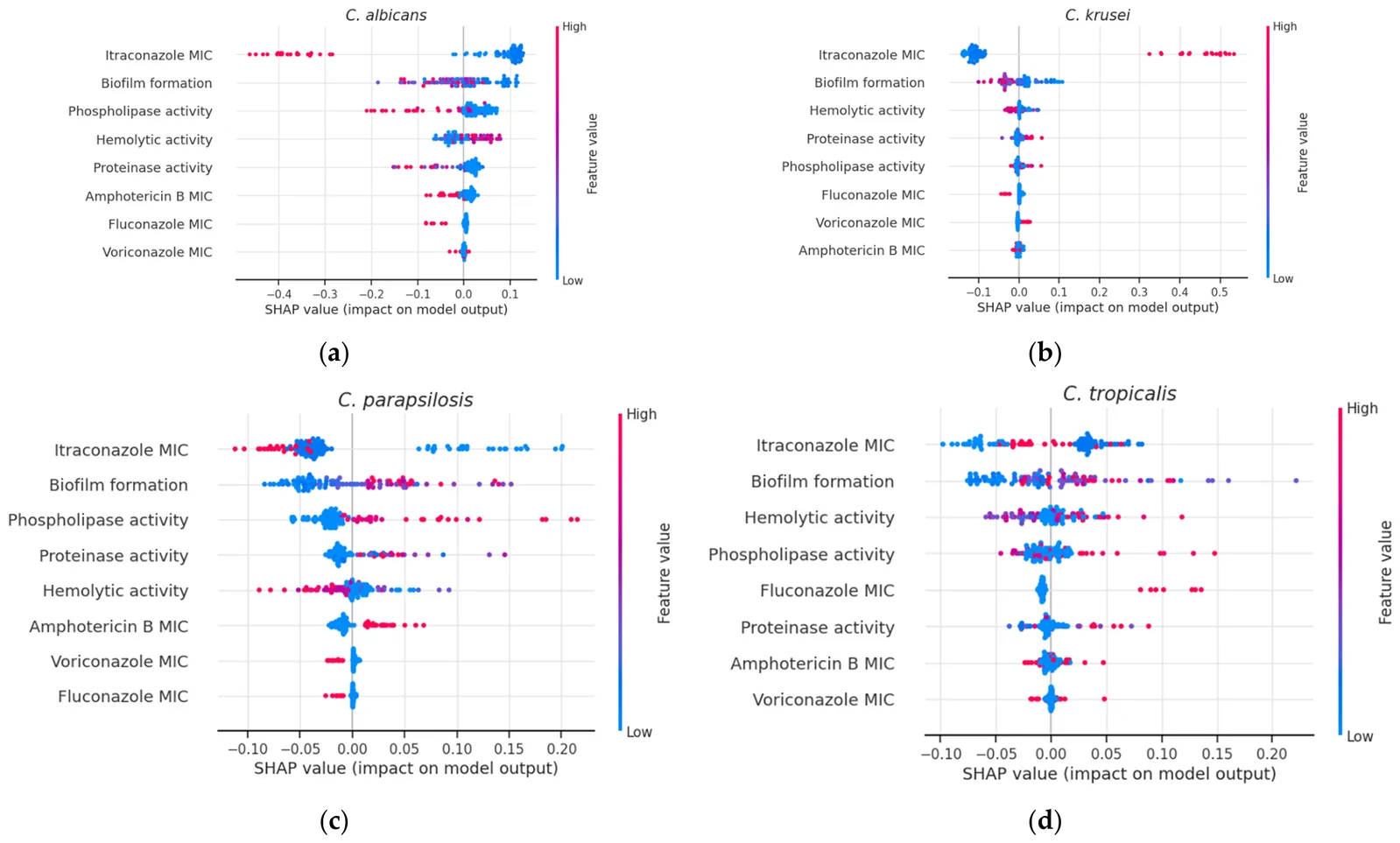
Species-specific SHAP summary plots for the Random Forest classifier: (**a**) *Candida albicans*; (**b**) *Candida krusei*; (**c**) *Candida parapsilosis*; (**d**) *Candida tropicalis*. Each point represents an individual isolate. Positive SHAP values indicate an increased probability of assignment to the respective species, whereas negative values indicate a decreased probability. Point color reflects the normalized feature value, ranging from low (blue) to high (red).

**Table 1:** Species distribution of the *Candida* isolates.

№	*Candida* Species	Abs (*n*)	%
1	*C. albicans*	74	69.2
2	*C. krusei*	18	16.8
3	*C. tropicalis*	8	7.5
4	*C. parapsilosis*	7	6.5
	Total	107	100

**Table 2:** Classification of biofilm formation according to Stepanović et al. (2007) [35].

Species (*n*)	No Biofilm, *n* (%)	Weak, *n* (%)	Moderate, *n* (%)	Strong, *n* (%)
*C. albicans* (*n* = 74)	41 (55.4)	24 (32.4)	9 (12.2)	0
*C. krusei* (*n* = 18)	14 (77.8)	3 (16.7)	1 (5.6)	0
*C. tropicalis* (*n* = 8)	2 (25.0)	4 (50.0)	2 (25.0)	0
*C. parapsilosis* (*n* = 7)	1 (14.3)	5 (71.4)	1 (14.3)	0
Total	58 (54.2)	36 (33.6)	13 (12.1)	0

**Table 3:** Species-specific antifungal susceptibility.

Species	Drug	MIC Range	MIC_50_	MIC_90_	S/WT% (*n*)	SDD% (*n*)	R/NWT % (*n*)
*C. albicans*	Fluconazole	1–4	2	2	97.2% (72/74)	2.7% (2/74)	0
	Voriconazole	0.06–0.125	0.06	0.125	87.8% (65/74)	12.2% (9/74)	0
	Itraconazole	0.5–4	1	1	-	-	-
	Amphotericin B	0.5–1	0.5	1	100% (74/74)	0	0
*C. tropicalis*	Fluconazole	2–4	2	2	75% (6/8)	25% (2/8)	0
	Voriconazole	0.06–0.125	0.06	0.06	87.5% (7/8)	12.5% (1/8)	0
	Itraconazole	1–4	1	4	-	-	-
	Amphotericin B	0.5–1	0.5	0.5	100% (8/8)	0	0
*C. parapsilosis*	Fluconazole	2	2	2	100% (7/7)	0	0
	Voriconazole	0.06	0.06	0.06	100% (7/7)	0	0
	Itraconazole	0.5–1	0.5	0.5	-	-	-
	Amphotericin B	0.5–1	0.5	1	100% (7/7)	0	0
*C. krusei*	Fluconazole *	-	-	-	-	-	-
	Voriconazole	0.06–0.125	0.06	0.125	-	-	-
	Itraconazole	4	4	4	-	-	-
	Amphotericin B	0.5–1	0.5	1	100% (18/18)	-	-

Note: S, susceptible; SDD, susceptible dose-dependent; R, resistant; MIC, minimum inhibitory concentration. Clinical breakpoints from CLSI M60 were applied to fluconazole and voriconazole. * *C. krusei* is intrinsically resistant to fluconazole. Amphotericin B isolates were wild-type (WT) according to CLSI M59 ECVs (≤2 µg/mL), reported here descriptively as susceptible. Itraconazole lacks established clinical breakpoints in CLSI M60; raw MIC values were utilized for analyses.

**Table 4:** Distribution of enzymatic virulence factors among *Candida* species.

Species	Phospholipase *n* (%)	Hemolysin *n* (%)	Proteinase *n* (%)
*C. albicans* (*n* = 74)	21 (28.4)	40 (54.1)	19 (25.7)
*C. krusei* (*n* = 18)	5 (27.8)	9 (50.0)	6 (33.3)
*C. parapsilosis* (*n* = 7)	4 (57.1)	4 (57.1)	3 (42.9)
*C. tropicalis* (*n* = 8)	2 (25.0)	4 (50.0)	3 (37.5)
*p*-value	0.327	0.762	0.643

**Table 5:** Phenotypic characteristics of the three identified phenotypic clusters of clinical *Candida* isolates.

Value	Cluster I, *n* = 63	Cluster II, *n* = 13	Cluster III, *n* = 31	H	*p*
Phospholipase score	0.000 (0.000–0.000)	0.000 (0.000–0.000)	0.455 (0.400–0.509)	95.73	<0.001
Proteinase score	0.000 (0.000–0.062)	0.000 (0.000–0.062)	0.000 (0.000–0.056)	0.16	0.923
Hemolysis score	0.067 (0.000–0.200)	0.200 (0.062–0.250)	0.000 (0.000–0.218)	2.73	0.256
Biofilm score	0.303 (0.219–0.385)	0.270 (0.212–0.449)	0.359 (0.239–0.524)	2.00	0.367
IVS	0.110 (0.078–0.145)	0.126 (0.113–0.185)	0.249 (0.196–0.301)	42.11	<0.001
Itraconazole MIC	1.000 (0.500–1.000)	1.000 (1.000–1.000)	1.000 (0.500–1.000)	2.86	0.240

Note: *p*—Kruskal–Wallis test.

**Table 6:** Pairwise comparisons among phenotypic clusters using Dunn’s test with Holm correction.

Value	I vs. II	I vs. III	II vs. III
Phospholipase score	0.350	<0.001	<0.001
Proteinase score	1.000	1.000	1.000
Hemolysis score	0.329	0.986	0.329
Biofilm score	0.972	0.473	0.972
IVS	0.156	<0.001	0.005
Itraconazole MIC	0.308	0.737	0.308

**Table 7:** Main characteristics of the identified phenotypic clusters of *Candida* isolates.

Phenotypic Cluster	% (*n*)	Main Phenotypic Profile
Cluster I	58.9 (63)	Low phospholipase activity and the lowest IVS
Cluster II	12.1 (13)	Low phospholipase activity and low-to-intermediate IVS
Cluster III	29.0 (31)	High phospholipase activity and the highest IVS

**Table 8:** Species composition of identified phenotypic clusters.

Species	Phenotypic Cluster I	Phenotypic Cluster II	Phenotypic Cluster III	*p*
*C. albicans*	46 (73.0)	9 (69.2)	19 (61.3)	-
*C. krusei*	9 (14.3)	3 (23.1)	6 (19.4)	-
*C. parapsilosis*	3 (4.8)	0	4 (12.9)	0.648
*C. tropicalis*	5 (7.9)	1 (7.7)	2 (6.5)	-

**Table 9:** Classification performance of the Random Forest model across 20 repeated 5-fold stratified cross-validation runs (100 validation folds total).

Performance Metric	Aggregated Out-of-Fold Performance	Mean ± SD	Empirical 95% Interval	Baseline
Accuracy	0.785	0.793 ± 0.017	0.757–0.818	0.692
Balanced accuracy	0.527	0.518 ± 0.030	0.461–0.577	0.250
Macro-F1	0.506	0.504 ± 0.031	0.444–0.559	0.204
Weighted F1	0.765	0.768 ± 0.015	0.740–0.793	–

Note: Baseline corresponds to a zero-rule majority classifier assigning all samples to *C. albicans*. 95% Empirical intervals were derived from empirical 2.5% and 97.5% percentiles across 20 independent repetitions of 5-fold stratified cross-validation.

**Table 10:** Class-specific performance metrics of the Random Forest classifier for the four *Candida* species.

Species	*n*	Precision	Recall	F1-Score	Correctly Classified, *n*
*C. albicans*	74	0.855	0.878	0.867	65
*C. krusei*	18	0.810	0.944	0.872	17
*C. parapsilosis*	7	0.286	0.286	0.286	2
*C. tropicalis*	8	0.000	0.000	0.000	0

## Data Availability

An anonymized minimal dataset supporting the statistical and machine-learning analyses in this study was submitted at the Editorial Office request.

## Abbreviations

The following abbreviations are used in this manuscript:

AI	Artificial Intelligence
BSA	Bovine Serum Albumin
CBP	Clinical Breakpoint
CI	Confidence Interval
CLSI	Clinical and Laboratory Standards Institute
COVID-19	Coronavirus Disease 2019
ECV	Epidemiological Cutoff Value
FDR	False Discovery Rate
Hz	Hemolytic Index
IQR	Interquartile Range
IVS	Integrated Virulence-related Score
MALDI-TOF MS	Matrix-Assisted Laser Desorption/Ionization Time-of-Flight Mass Spectrometry
MIC	Minimum Inhibitory Concentration
MOPS	3-(N-morpholino)propanesulfonic acid
NAC	Non-*albicans Candida*
NWT	Non-Wild Type
OD	Optical Density
ODc	Optical Density Cut-off
PBS	Phosphate-Buffered Saline
PCA	Principal Component Analysis
PCR	Polymerase Chain Reaction
PERMANOVA	Permutational Multivariate Analysis of Variance
Prz	Proteinase Index
Pz	Phospholipase Index
RF	Random Forest
RPMI 1640	Roswell Park Memorial Institute 1640 medium
SD	Standard Deviation
SDD	Susceptible Dose-Dependent
SHAP	SHapley Additive exPlanations
SVM	Support Vector Machine(s)
UMAP	Uniform Manifold Approximation and Projection
WT	Wild Type
